# Supplementary figures and images for: H2O2 gel bleaching induces cytotoxicity and pain conduction in dental pulp stem cells via intracellular reactive oxygen species on enamel/dentin disc
Source: PLoS One. 2021 Sep 10;16(9):e0257221. doi: 10.1371/journal.pone.0257221 (PMC8432789; doi:10.1371/journal.pone.0257221)

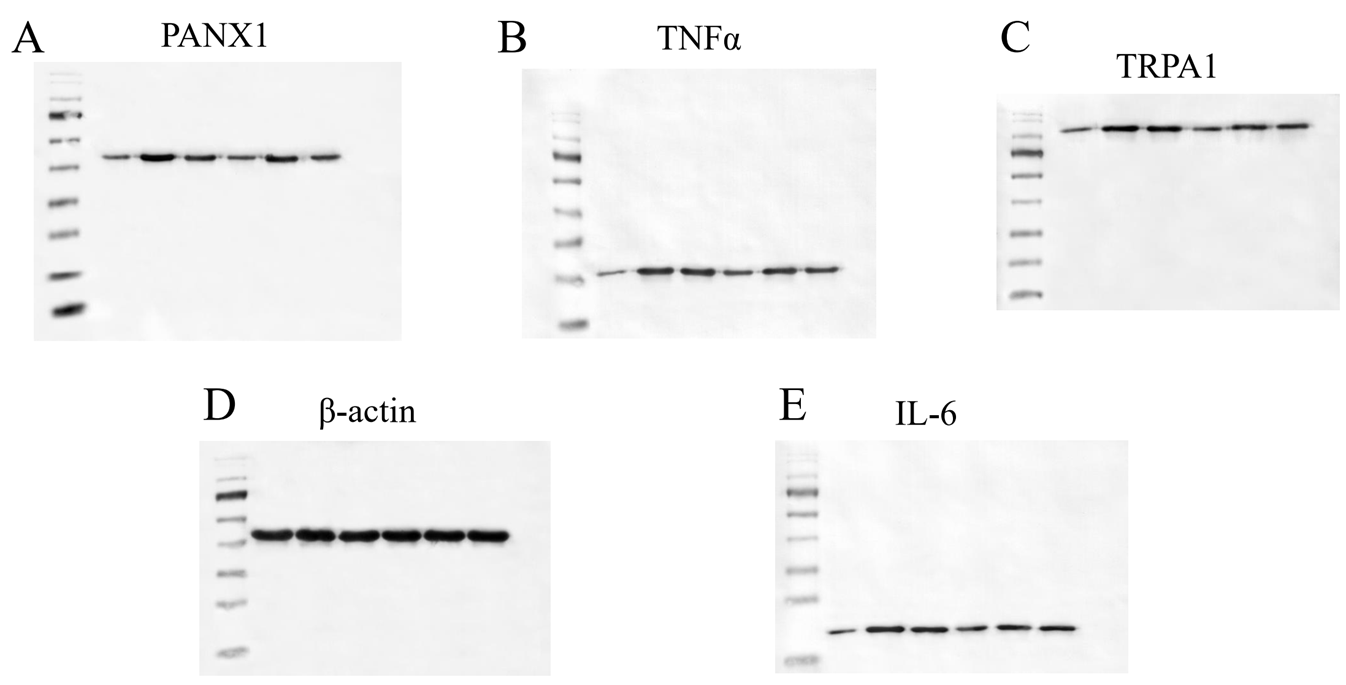

Supplement: S1 Fig — (A) PANX1. (B) TNFα. (C) TRPA1. (D) β-actin. (E) IL-6. (TIF) [file pone.0257221.s001.tif]
