# Supplementary material for: H2O2 gel bleaching induces cytotoxicity and pain conduction in dental pulp stem cells via intracellular reactive oxygen species on enamel/dentin disc
Source: PLoS One. 2021 Sep 10;16(9):e0257221. doi: 10.1371/journal.pone.0257221 (PMC8432789; doi:10.1371/journal.pone.0257221)
Supplement: Attachment — Submitted filename: Response_to_Reviewers.docx [file pone.0257221.s003.docx]

Dear Gianpaolo Papaccio, M.D., Ph.D.,

Thank you very much for your letter and further instructions. We also appreciate the reviewers’ valuable and constructive comments concerning our manuscript, entitled “**H_2_O_2_ gel bleaching induces cytotoxicity and pain conduction in dental pulp stem cells via intracellular reactive oxygen species on enamel/dentin disc**” (PONE-D-21-18951). After careful consideration of the comments, we have extensively revised our manuscript, as indicated by the tracked changes. Our point-by-point responses are provided below.

For journal requirements:

1. We have modified the our manuscript according to PLOS ONE's style requirements.
2. We have added information on animal experiments as required and submitted the ARRIVE Guidelines checklist.
3. We apologize for using the western image that was acquired after cutting the membrane. In order to provide the entire original image without trimming, we repeated the western experiment and updated the blots of Figures 3 and 4. The original picture is shown in the additional material Figure s1.
4. We have linked ORCID iD in the revision.

Sincerely,

Rong Li

Department of Stomatology, The Second Xiangya Hospital, Central South University, Changsha, Hunan, China.

E-mail: [rongli@csu.edu.cn](mailto:rongli@csu.edu.cn)

Responses to the reviewers’ comments:

1. Introduction section should be reduced.

**Response:** Thank you for your careful review. In order to shorten the length of the introduction section, we have reduced the word count from 632 to 517.

2. Authors should add other experiments to evaluate ROS production in DPSCs (Cell-Rox assay).

**Response:** Thank you for your comment. We have added the Cell-Rox assay (Fig 4C and D). (line 165)

3. Evaluation of in vivo dental pulp after blenching treatments should be made also at longer times (up to 30 days).

**Response:** Thank you very much for your suggestion. We have added 30 days result in the revised manuscript (Figures 5, 6, and 7).

4. In discussion section Authors should emphasize the role of DPSCs (BioMed Research International, 2021; Comprehensive Biomaterials, 2011)

**Response:** Thank you for your suggestion. We have added the role of DPSCs in the second paragraph of the discussion section. (lines 361-371)
